# Determination of effective half-life of ^131^I in thyroid cancer patients using remote dose-rate meter

**DOI:** 10.1186/s40658-024-00701-8

**Published:** 2024-11-29

**Authors:** Laura Kääriä, Maria Lapela, Marko Seppänen, Mikael Högerman, Johanna Ruohola, Annika Ålgars, Tommi Noponen

**Affiliations:** 1 Department of Clinical Physiology, Nuclear Medicine, Turku PET Centre, and Medical Physics, Turku University Hospital and Wellbeing services county of Southwest Finland and University of Turku, Turku, Finland; 2 Department of Oncology, Turku University Hospital and Wellbeing services county of Southwest Finland, Turku, Finland; 3 Department of Clinical Physiology, Nuclear Medicine, and Turku PET Centre, Turku University Hospital and Wellbeing Services County of Southwest Finland, Turku, Finland; 4 Department of Urology, Department of Mathematics and Statistics, Turku University Hospital and Wellbeing services county of Southwest Finland and University of Turku, Turku, Finland; 5 Department of Clinical Physiology, Nuclear Medicine, Turku PET Centre, and Medical Physics, Turku University Hospital and Wellbeing Services County of Southwest Finland, Turku, Finland

**Keywords:** ^131^I, Radioactive iodine, Effective half-life, Radiation safety, Remote dose-rate meter

## Abstract

**Background:**

Continuously monitored external dose-rate signals from remote dose-rate meters (DRMs) were analyzed to determine the effective half-life (T_eff_) of ^131^I in differentiated thyroid cancer (DTC) patients. The aim is to gain novel understanding of the excretion of radioactive iodine (RAI) in DTC patients and to demonstrate that a remote DRM system can be reliably used for real-time monitoring of external dose-rates of DTC patients.

**Methods:**

110 DTC patients who received postoperative RAI therapy between September 2018 and February 2023 in Turku University Hospital were studied retrospectively. The external dose-rates of the patients were continuously monitored during their hospitalization with a remote DRM fixed in the ceiling of the isolation room. Generalized linear mixed model (GLMM) was used to analyse the association between logarithmical T_eff_ and patient characteristics.

**Results:**

The median T_eff_ for all patients was 12.60 h (Q1: 10.35; Q3: 14.75 h). Longer T_eff_s were associated with higher BMI (*p* = 0.004), lower GFR (*p* < 0.001), and diabetes (*p* = 0.007). Our study also revealed that neither age nor subsequent RAI therapies have a significant impact on the whole body T_eff_ (*p* = 0.522 and *p* = 0.414, respectively).

**Conclusion:**

Patients with higher BMI, decreased GFR, or diabetes have a longer whole-body T_eff_ of ^131^I. Ceiling-mounted remote DMRs can reliably be used to determine patient’s T_eff_. Since T_eff_ values vary among patients, ceiling-mounted meters can be used to optimize the length of radiation isolation period at the hospital while improving patient comfort and staff efficiency.

## Introduction

Radioactive iodine (RAI) therapy implemented using ^131^I nuclide is an efficient and well-tolerated method for the treatment of differentiated thyroid cancer (DTC) after total thyroidectomy for selective irradiation of thyroid remnants, microscopic disease, and to prevent cancer recurrence [1, 2]. In recent years, the incidence of DTC has increased due to advancements in imaging modalities enabling early detection of the disease, with fewer subclinical tumors remaining indiscernible [2, 3]. While this increase is also attributed to overdiagnosis of the disease, the demand for RAI therapy may increase in the future as the number of patients is increasing globally [3].

Due to high-energy gamma emission, relatively long physical half-life, and multiple excretion routes from the body, radiation protection precautions for ^131^I are usually more rigorous compared to other therapeutic radiopharmaceuticals [4, 5]. RAI therapy poses a potential radiation risk to other people and the environment due to possibility of external and internal radiation exposure. ICRP and IAEA have recommended 1 mSv annual dose limit for general public that is prescribed by law in many countries [6]. To minimize the radiation exposure to family members and other individuals, many countries require a few days of radiation isolation in the hospital after RAI therapy [7–9].

The length of radiation isolation is highly dependent on the excretion rate of the activity in the patient. Realistic information on the clearance of a radiopharmaceutical from patient’s body can be described with effective half-life (T_eff_), which is a combination of biological and physical half-lives [10]. Although the mean value of T_eff_ does not individually represent all the patients, its determination benefits the recognition of the patients’ clinical characteristics that may affect on the ^131^I excretion rate from the body. Precise knowledge of the clearance of ^131^I facilitates planning the needed hospitalization length as well as individualizing radiation safety precautions for patients [8, 11, 12].

The external dose-rate of the patient should be measured at least once before the patient is discharged from the hospital, preferably daily [2]. However, daily dose-rate measurements may unnecessarily expose medical staff to radiation since it is traditionally done by using a hand-held meter. Ceiling-mounted remotely operated dose-rate meter (DRM) can be used to monitor patients’ external dose-rate levels continuously during hospitalization without exposing the staff [13, 14].

The aim of this study is to gain novel understanding of the excretion of ^131^I in DTC patients by investigating whether there is a relationship between patients’ clinical characteristics and the T_eff_ of ^131^I. We also demonstrate that a ceiling-mounted remote DRM can be reliably used in the real-time monitoring of DTC patients’ external dose-rate levels during hospitalization.

## Materials and methods

### Study population

The study cohort consists of 110 DTC patients (74 females and 36 males) aged between 14 and 85 years who underwent postoperative RAI therapy between September 2018 and February 2023 in Turku University Hospital, Finland. Two patients who received RAI during this period, while remote DRMs were in maintenance, were excluded. According to the clinical practice all patients had previously undergone total thyroidectomy.

### Pathology and laboratory tests

Histological status of cancer was determined from pathologic-anatomical diagnosis (PAD) along with the presence of possible lymph node metastasis in the sample. Additionally, the tumor characteristics were also investigated. TNM-staging was done for the majority of patients based on AJCC/UICC staging system [15]. Patients were referred to RAI ablation based on the stage classification and medical eligibility according to clinical practice. The medical history of the patients were also reviewed.

Laboratory tests including thyroid-stimulating hormone (TSH), thyroglobulin (TG), serum creatinine (sCr), and glomerular filtration rate (GFR) were done prior ablation.

### Patient preparation and RAI therapy

Patients adhered to a low-iodine diet for two weeks. Due to the coronavirus outbreak in 2020, the iodine-deficient diet for two patients was shortened by a few days. TSH stimulation (> 30 mU/l) was achieved with two thyrotropin alpha (Thyrogen^®^) injections, administered 24 and 48 h before the RAI treatment. TSH and serum TG were monitored the same morning before to ensure successful TSH stimulation level for the ablation and TG for a reference value for patient follow-up.

Patients were given oral and written information regarding the progress of RAI treatment in the department and radiation protection during hospitalization and after discharge from the hospital. In Finland, standardized activities of 1.11 GBq and 3.70 GBq were employed, according to the cancer characteristics and the risk of its recurrence [16, 17]. For subsequent treatments, a 3.70 GBq activity of RAI was employed.

All patients were hospitalized in one of our two radiation-protected isolation rooms typically from 1 to 3 days [6, 16]. The patients were discharged from the hospital when their external dose-rate was less than 15 µSv/h measured at a distance of one meter with a hand-held DRM [8]. The patient may also be discharged, if the measured dose-rate is between 15 and 40 µSv/h at the discretion of medical physicist, along with individual tightened radiation protection instructions. The discharge limit was chosen to fulfil national dose limits for family members and individuals [18].

### Cloud-based remote dose-rate meters

The whole-body T_eff_ of patient was determined by measuring the external dose-rate acquired with one of the two remote cloud-based DRMs (Sensire Dosimeter, Sensire, Finland) containing KATA DGM-1500 Turva DRM (KATA, Finland) that utilizes an ambient dose equivalent-energy compensated Geiger-Müller counter [19]. DRMs were installed in the ceilings of both isolation rooms, located approximately two meters above the hospital bed, continuously monitoring the dose-rate of their surroundings.

The data were collected at one-minute intervals, enabling the system to generate a nearly real-time graph of dose-rates as a function of time. To achieve individual patient records, the data were meticulously downloaded into separate .xlsx-files for each patient.

### Dose-rate meter calibrations

The measured dose-rate signals are affected by patient’s movements in the room during isolation as well as radiation attenuation by the body. To ensure more accurate readings, patients’ location and position changes were incorporated using correction factors (CFs) obtained through calibration with a moderate-activity (A = 485 MBq) point source, a ^131^I capsule. Remote DRMs have a reasonable linearity (± 10.0%) over a wide range of dose-rates (0.01–100,000 µSv/h), which allows the use of a less-radiation-toxic source for calibration.

Calibration measurements were executed at three different locations in both rooms, where patients were most likely to spend time during isolation, such as laying supine or sitting on the patient bed and sitting on a chair by a table (approx. 2.0 m, 2.1 m and 3.5 m from the remote DRM, respectively). Laser distance meter (Fluke 424D, Fluke Finland Oy, Finland) was utilized to determine the distances between the DRM and these three measurement points. Five time-points average dose-rate values were measured at each location for the final calculations. The CFs were calculated as a ratio of the dose-rate reading measured in the supine position on the bed (reference location) to that measured in one of two other calibration locations. Additionally, the radiation attenuation by the body was compensated in the sitting position on the bed by estimating the path of radiation through the patient body and utilizing an attenuation coefficient specific to ^131^I in the water. The attenuation correction was carried out only in the single position because the attenuation effect was approximated to be the same in two other calibration locations. CFs made possible to correct fluctuations in dose-rate signals due to changes in the patient’s location and position in the isolation room.

### Preprocessing of measurement signals

**Fig. 1:**
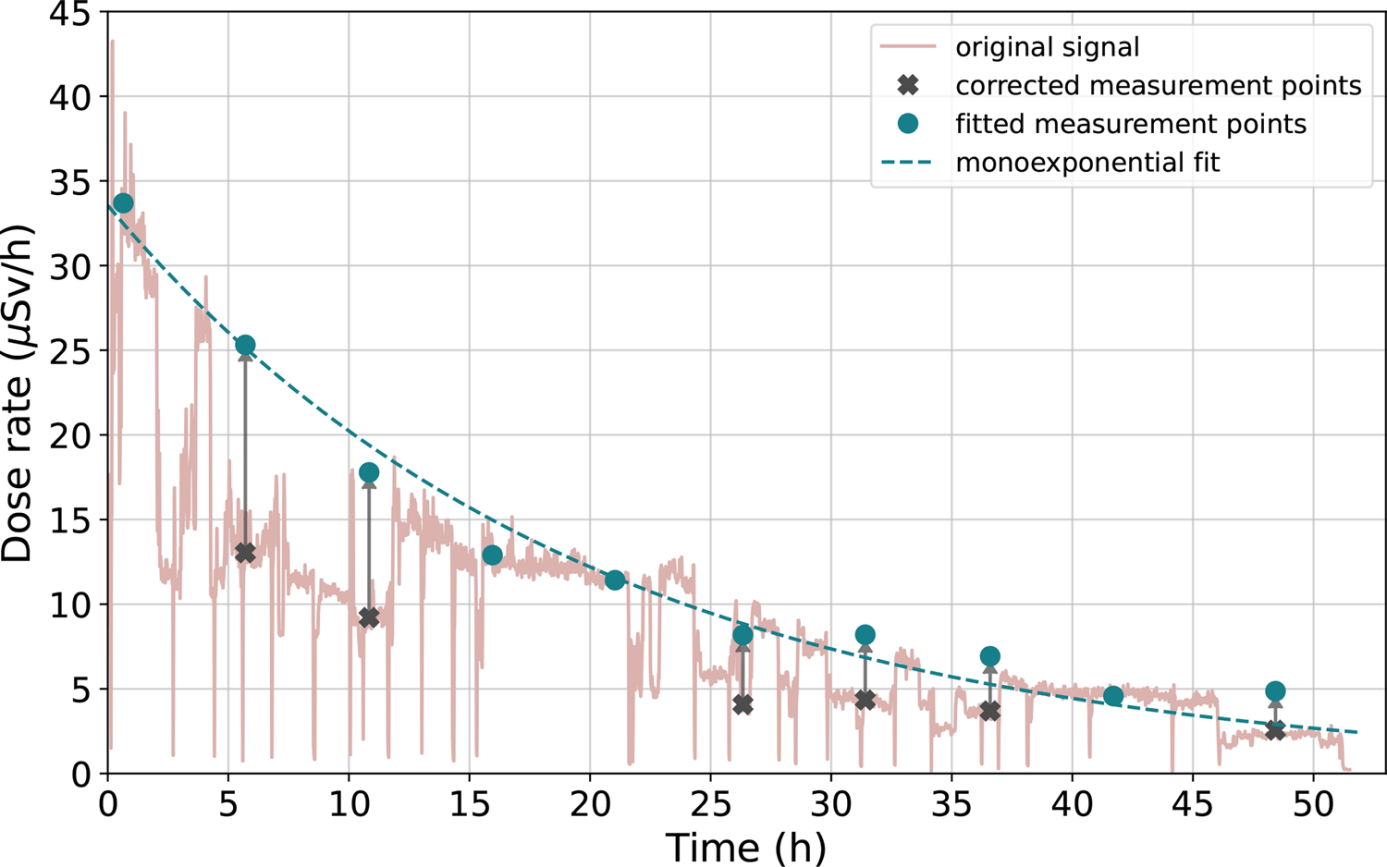
An example remote dose-rate signal from 2.0 m distance during the isolation of a patient who received 3.70 GBq RAI. Original signal from the remote DRM is shown with light pink color. Before fitting a monoexponential function to measurement points (blue dots), some of the dose-rate values (black ticks) were corrected using the location CFs

To ascertain T_eff_ of patients, ten measurement points were extracted from each patient’s dose-rate signal. The initial measurement point was selected within the first ten to thirty minutes after RAI administration. Subsequent measurement points were sampled evenly throughout the isolation period, from administration to discharge, providing a comprehensive representation of the clearance process. Due to the retrospective nature of our study, 13.8% of the sampled data points were corrected afterwards. Two CFs (1.97 for short distance and 2.10 for long distance) defined based on the calibration procedure described above were employed to adjust the dose-rate values. Background dose-rate was subtracted before curve fitting. Figure 1 visualizes the use of CFs in practice. To determine the T_eff_, a monoexponential function was fitted to the selected measurement points. The T_eff_ was calculated from the fitted equation.

### Statistical analysis

The goodness of the monoexponential fits were evaluated by R-squared values (R^2^) that were calculated with Microsoft Excel 2016 v. 16.0 (Microsoft Office, Microsoft Corporation, USA). One-dimensional K-means clustering analysis was performed to identify similarities within the distribution of the T_eff_ values. The clustering analysis was performed using Python v. 3.10 with three predefined clusters.

Logarithmical (Log) transformation was used to transform T_eff_ data to conform normality. Normality assumptions were then evaluated visually using histograms and quantile-quantile (Q-Q) plot with standardized residuals of logarithmic values of T_eff_. Generalized linear mixed model (GLMM) was used to analyze the relationship between Log T_eff_ and the original 26 patient characteristics. Parameters that were known to correlate with each other or were calculated based on another parameters, such as GFR and sCr, were excluded from the extensive GLMM analysis. The reduced GLMM model was constructed with parameters known to be significant based on the scientific literature and by removing one-by-one the least significant parameters based on the obtained p-values [9–11, 20–26]. The final GLMM model consisted of the seven most significant parameters: age, body mass index (BMI), GFR, other comorbidities, TG, administered activity, and treatment cycle. Statistical significance level was set at 0.05 and 95% confidence intervals (CI) were calculated. Categorical variables were described in percentage proportions and continuous variables which followed normal distribution with mean and standard deviation (SD) and otherwise with median and first and third quartiles (Q1, Q3). The analyses were carried out using IBM SPSS software package (v. 29.0 SPSS Inc., IBM Company, USA).

## Results

A total of 135 external dose-rate signals of 110 DTC RAI therapy patients were analyzed retrospectively. Patient and disease characteristics are presented in Tables 1 and 2. Within this patient cohort, 72.6% had papillary histology, 26.7% follicular patterns, and one patient features of poorly differentiated thyroid carcinoma. This study includes 28 low-risk and 107 high-risk patient cases receiving 1.11 and 3.70 GBq treatment activity, respectively. Majority of the patients (*n* = 93) in the study group received a single treatment and 42 of the patients received 2–4 RAIs (26 two RAIs, 10 three RAIs, and 6 four RAIs). Some of the patients had been treated at least once before this study period and some had received their previous treatment(s) in another hospital.

**Table 1:** Clinical characteristics of thyroid cancer patients

Characteristic	Overall
Gender, n (%)	135 (100%)
Male	46 (34.1%)
Female	89 (65.9%)
Age (years), mean ± SD	55.21 ± 16.53
BMI (kg/m^2^), median (Q1, Q3)	27.17 (23.62, 31.74)
Administered activity (GBq), n (%), mean ± SD	
1.11 GBq	28 (20.7%), 1.13 ± 0.04
3.70 GBq	107 (79.3%), 3.77 ± 0.15
Effective half-life (hours), median (Q1, Q3)	12.60 (10.35, 14.75)
TSH (mU/l), median (Q1, Q3)	130 (84, 170)
TG (ug/l), median (Q1, Q3)	5.6 (0.6, 21.7)
TG Abs^a^ (kU/l), median (Q1, Q3)	< 20 (< 20, < 20)
sCr (umol/l), median (Q1, Q3)	72 (63, 84)
GFR (ml/min/1.73 m^2^), mean ± SD	87 ± 19

BMI, body mass index; TSH, thyroid-stimulating hormone; TG, thyroglobulin; TG Abs, thyroglobulin antibodies; sCr, serum creatine; GFR, glomerular filtration rate

^a^TG Abs level was under < 20 kU/l for the majority of the studied patients

**Table 2:** Disease characteristics of thyroid cancer patients

Disease characteristics					
	*Papillary*		*Follicular*	*Poorly differentiated*	
Histology, n (%)	98 (72.6%)		36 (26.7%)	1 (0.7%)	
Primary tumour volume cm^3^, median (Q1, Q3)	6.49 (1.53, 33.51)				
Number of tumours, median (Q1, Q3)	1 (1, 2)				
	*Yes*	*No*	*Uncertain*	*Missing*	
Tumour capsule, n (%)	74 (54.8%)	36 (26.7%)	22 (16.3%)	3 (2.2%)	
Invasion through tumour capsule, n (%)	55 (40.7%)	44 (32.6%)	33 (24.4%)	3 (2.2%)	
Lymphovascular invasion (LVI) , n (%)	35 (25.9%)	70 (51.9%)	27 (20%)	3 (2.2%)	
Invasion outside thyroid, n (%)	22 (16.3%)	71 (52.6%)	42 (31.1%)	N/A	
Growth through lymph node capsule, n (%)	8 (5.9%)	30 (22.2%)	N/A	97 (71.9%)	
Metastasized disease, n (%)	66 (48.9%)	47 (34.8%)	22 (16.3%)	N/A	
	*T1*	*T2*	*T3*	*T4*	*Missing*
Tumour, n (%)	34 (25.2%)	37 (27.4%)	15 (11.1%)	13 (9.6%)	36 (26.7%)
	*N0*	*N1*	*Nx*	*Missing*	
Node, n (%)	47 (34.8%)	42 (31.1%)	12 (8.9%)	34 (25.2%)	
	*M0*	*Mx*	*Missing*		
Metastasis, n (%)	52 (38.5%)	25 (18.5%)	58 (43.0%)		
	*Neck lymph node*	*Neck area*	*Distant metastasis*	*Missing*	
Site of metastases, n (%)	40 (29.6%)	8 (5.9%)	16 (11.9%)	71 (52.6%)	
	*1*	*2*	*3*	*4*	
Treatment cycle, n (%)	93 (68.9%)	26 (19.3%)	10 (7.4%)	6 (4.4%)	
	*Cardiovascular diseases*	*Diabetes*	*Other malignancy*	*Other/non*	*various diseases*
Other comorbities, n (%)	31 (23.0%)	7 (5.2%)	6 (4.4%)	82 (60.7%)	9 (6.7%)

The mean and standard deviation (SD) of the R^2^ values of the fitted monoexponential functions were 0.96 ± 0.05. Therefore, the model for effective whole-body decay of RAI seems to be monoexponential.

The median whole-body T_eff_ of ^131^I for all patients was 12.60 h (Q1: 10.35; Q3: 14.75) (Table 1). No statistically significant difference (*p* = 0.171, Table 3) was observed in T_eff_ values between patients who received 1.11 and 3.70 GBq. The administered activity in 28 low-risk patient cases varied from 1.06 to 1.22 GBq, while in 107 high-risk patient cases between 3.15 and 4.05 GBq.

**Table 3:** Reduced GLMM for variables predicting T_eff_

	Coefficient^a^ (95% CI)	SE	*p* value*
Age	-0.999 (0.995–1.003)	1.102	0.522
BMI	1.011 (1.003–1.018)	1.133	0.004
GFR	-0.994 (0.991–0.997)	1.143	< 0.001
Other comorbidities	-	-	0.001
Cardiovascular diseases	-0.97 (0.803–1.18)	1.098	0.786
Diabetes	1.41 (1.102–1.803)	1	0.007
Other malignancy	-0.96 (0.736–1.249)	1.004	0.752
Other/non	-0.89 (0.738–1.069)	1.002	0.206
Various diseases	0^b^ (1–1)	1	-
TG	-1 (1–1)	1	0.103
Administered activity	0.97 (0.929–1.013)	1.022	0.171
Treatment cycle	-	-	0.414
1. RAI	0.91 (0.743–1.12)	1.109	0.376
2. RAI	0.87 (0.697–1.075)	1.116	0.19
3. RAI	0.98 (0.763–1.262)	1.135	0.881
4. RAI	0^b^	-	-

BMI, body mass index; GFR, glomerular filtration rate; TG, thyroglobulin; RAI, radioactive iodine

*Generalized linear mixed model

^a^. Coefficients indicate the impact of a 1-unit change in a predictor variable while others are held constant in the model

^b^. This redundant coefficient is set to zero

**Fig. 2:**
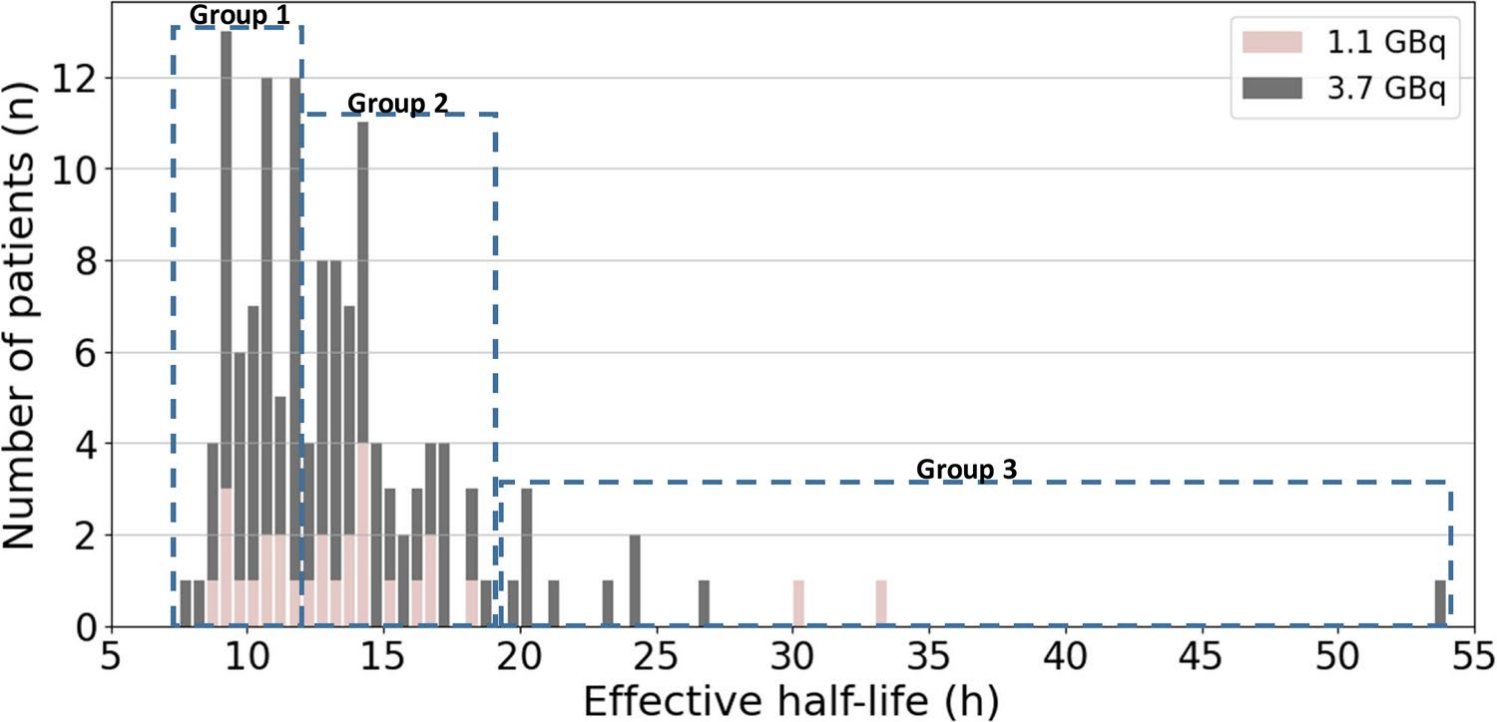
The distribution of T_eff_s of DTC patients. Light color bars represent the patients who received 3.70 GBq and dark color bars the patients who received 1.11 GBq activity. Dashed line rectangles indicate the division of T_eff_ s into three groups

In Fig. 2a histogram diagram of T_eff_ values is presented for the both administered activities. The group division by K-means clustering analysis is also shown in the figure. The median T_eff_ from the shortest half-life to longest in Groups 1–3 were 10.19 h (Q1: 9.12; Q3: 10.83 h), 14.00 h (Q1: 13.08; Q3: 15.49 h) and 23.50 h (Q1: 19.80; Q3: 29.27 h), respectively. Groups 1 and 2 express moderate level of homogeneity whereas Group 3 shows significant heterogeneity. Overall, the T_eff_ data appears highly right-skewed and a large variation in T_eff_ values is distinguished in the study population.

The effect of age, BMI, GFR, TG, other chronic comorbidities, administered activity and treatment cycle to T_eff_ is shown in Table 3. A significant association was observed between GFR and T_eff_ (*p* < 0.001), showing that a decreasing GFR is strongly associated with prolonged T_eff_. Additionally, an increasing BMI was linked to a prolonged T_eff_ (*p* = 0.004) along with other chronic comorbidities (*p* = 0.001), particularly diabetes.

**Fig. 3:**
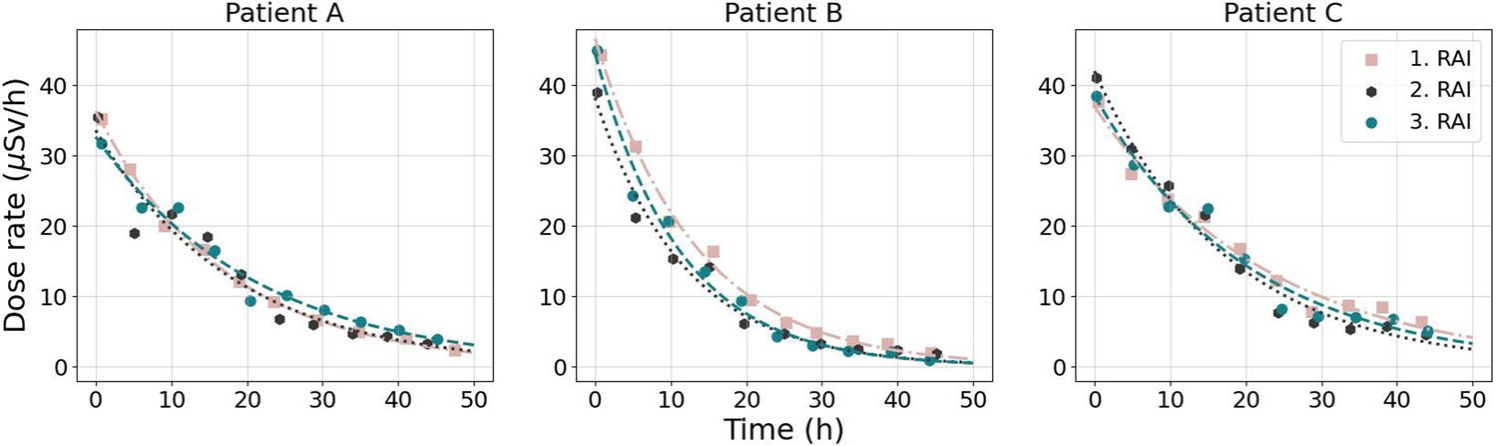
^131^I dose-rate curves for three DTC patients that have received three RAI treatments. Monoexponential functions fitted to the data points are shown with the color-coded dashed lines. All three patients received 3.70 GBq activity in each cycle

The T_eff_ does not significantly depend on the number of RAI treatment cycles (*p* > 0.1, Table 3). The impact of treatment cycles to the decay of RAI is further illustrated in Fig. 3 for three high-risk patients (A, B, and C). The T_eff_s for Patient A were 12.38, 12.60, and 15.07 h, for Patient B 9.76, 10.05, and 8.15 h, and for Patient C 16.91, 12.60, and 14.75 h for the first, second, and third RAI cycle, respectively.

## Discussion

In this study, we had a novel setup to investigate the T_eff_ of ^131^I in DTC patients. Continuous monitoring of patients’ external dose-rates with ceiling-mounted meters was conducted throughout their hospitalization, from treatment administration to the discharge from radiation isolation. The continuous measurement did not cause any inconvenience to the patients, which was not the case in the previous T_eff_ studies [9, 11, 26]. In addition, the utilization of remote DRMs does not risk the medical staff to any unnecessary radiation dose. However, it is crucial to ensure that the DRM stays constantly in place during clinical practice to guarantee accurate readings.

Our study showed that a ceiling-mounted DRM can reliably be used to determine the T_eff_ of ^131^I in DTC patients. The median value of T_eff_ 12.60 h (Q1: 10.35; Q3: 14.75 h) for all patients is consistent with previously reported values [9, 11, 24–26]. Previous studies have used either mono- or biexponential model to determine T_eff_ from measured dose-rate data. In our study the dose-rate data were collected during isolation period, mostly within the first 48 h after RAI administration. Throughout this period the removal of ^131^I is quite rapid, which allowed us to use the monoexponential fitting reliably to the data (R^2^ = 0.96 ± 0.05). Biexponential fitting would be more suitable for longer measurement periods such as for a week long period [8].

In our study population, longer T_eff_ values were associated with increasing BMI (*p* = 0.004), decreasing GFR (*p* < 0.001), and diabetes (*p* = 0.007). To our knowledge, this is the first study to show statistical significance between BMI and T_eff_ of ^131^I in DTC patients [9]. Since iodine is mainly eliminated through the urinary tract, it was assumed that GFR significantly affects the T_eff_ values in DTC patients. This is also shown in the previous studies where renal function has influenced significantly in the clearance time of the ^131^I activity [22, 23]. Diabetes has been previously mentioned to be associated with a prolonged T_eff_, moreover it is well known that diabetes and increased BMI are associated with decreased GFR [27–29]. To further elucidate a possible interaction, we investigated the interaction terms of these parameters on the T_eff_ in GLMM. However, interaction terms were not statistically significant, leading to their exclusion from the model. Furthermore, no statistical significance was observed between age and T_eff_ that is an opposite result to what has been reported in a recent study [9]. This could be explained with differences in the study populations or in the used study methodology.

K-means clustering analysis divided our patients into three groups based on similarities in determined T_eff_ values (Fig. 2). The distribution in Groups 1 and 2 appears to be quite similar. However, we were the most interested in patients in Group 3 associated with the longest T_eff_ in the study population. 55% of the patients with diabetes in our study cohort belonged to Group 3. 50% of them had also decreased renal function with a low GFR value, although only one of them had a diagnosed renal failure. Also 67% of patients in Group 3 were overweight or obese and 62.5% of them were extremely obese (BMI > 35). This further indicates that nonspecific accumulation of ^131^I, not trapped in thyroid remnant tissues but rather circulating in the body, has decreased removal rate in patients with a high BMI and diabetes as well as in patients with a low GFR value.

In lesion-based studies subsequent RAI therapy cycles have been shown to decrease the T_eff_ values of ^131^I in DTC lesions [21]. The T_eff_ in lesions has been found to depend on thyroid remnant mass as well as on the ability of the remaining thyroid cells to uptake and store ^131^I [20, 26]. A debated phenomenon known as the self-stunning effect, induced by the administration of ablative doses of ^131^I, may diminish a capability to uptake RAI in thyroid remnant tissues [30, 31]. According to current knowledge, the stunning effect influences only on the RAI uptake in the remnant tissue in the short term, typically during the first hours of the RAI administration which should not observably affect on the whole-body T_eff_ [32].

The impact of thyroid remnant mass on T_eff_ of ^131^I is however evident. In situations where patients have evidence of a significant amount of thyroid remnant tissue most often seen in pyramidal lobe due to challenges in total thyroidectomy, a large amount of the administered RAI can be trapped in that tissue. Based on minor modifications done by Johansson et al. to the IRCP 56/67 biokinetic model the biological half-life of iodine in adults’ thyroid tissue is 80 days [33]. Patients with less remnant tissue, less RAI is trapped in such tissue when compared to patients with more remnant tissue. In this study the longest T_eff_ value was 53 h which was partly caused by an incomplete thyroidectomy. The impact of challenges in performing thyroidectomy on T_eff_ of ^131^I has also been previously highlighted [11].

It was unexpected that repeated RAI cycles do not significantly affect on the T_eff_ of ^131^I at least during the radiation isolation period. Subsequent RAI therapies are programmed if TG level is high, it increases during the follow-up, or for treatment of RAI-avid metastases. Since the impact of subsequent therapies on the T_eff_ is evident in the lesion-based studies, this might not be the case when studying whole-body T_eff_ [21]. Although remnant mass is reduced due to ablation treatments, patient’s other physiological characteristics seem to have more dominant impact on T_eff_, at least during the hospitalization period (Table 3; Fig. 3).

This study was performed retrospectively, which means the patients were not given any guidance regarding the study protocol and the external dose-rates were continuously monitored. Measuring setup was modelled afterwards when patient’s locations were corrected with two CFs to standardize the locations into the hospital bed. In previous studies, patients followed a specific protocol in which they went in the specific measurement location at the certain time intervals [9, 11]. Often patients were also advised to empty their bladder prior measurements, with the exception of the first measurement that was taken right after administration. The remaining activity in the bladder significantly affects the patient’s external dose-rate, as RAI is mostly eliminated through urinary tract. Our patients were advised as part of our normal radiation protection routines to drink preferably a glass of water or other liquids once in an hour and empty their bladder always when needed during the isolation to enhance the removal of excess RAI from the body. No further instructions which significantly affect on the T_eff_ of ^131^I were given. Therefore, in our study the T_eff_ values may have been defined more realistically than in the previous studies since our patients did not have any protocol related restrictions during their isolation period.

It is possible that the used CFs might slightly overestimate patient’s dose-rate especially if applied during the first four hours. The thickness of radiation absorbing tissue may also vary widely among the patients, mostly due to differences in body size. These technical limitations may have slightly compromised our accuracy to determine the exact T_eff_ values. The CFs were, however, applied only to minor number of data points (~ 13.8%) and the R^2^ of the fitted monoexponential functions had relatively high values (0.96 ± 0.05) showing that the location and attenuation corrections seemed to work well.

Our study identified an association between increased BMI and the T_eff_ of ^131^I, which could be further investigated with a larger patient cohort, ideally including patients with a wider range of BMIs. In this study, patients received instructions regarding fluid intake in a general level. It would be interesting to explore whether more detailed instructions regarding fluid intake would impact the T_eff_ of ^131^I. Differences in radiopharmaceutical distributions due to differences in metabolic rates in patients may have an influence on the measurement accuracy of T_eff_ values. However, these differences did not affect our T_eff_ results crucially since the exponential curve fittings worked reasonable well in this study. Additionally, the significance of the tumour burden on patients’ T_eff_ could be determined by calculating quantitative values from SPECT/CT images to understand their effect on the T_eff_. In the future, it would also be interesting to compare the dose-rate readings from the manual hand-held and remote DRMs. The remote DRMs’ software user interface could also be further improved by incorporating CFs, allowing for prospective correction of dose-rate signals.

Since T_eff_ varies among patients, it is unfeasible to provide uniform safety instructions to patients after hospital discharge. Remote dose-rate monitoring provide an effective technical solution to measure these T_eff_ differences. Beyond variations in ^131^I clearance, patient circumstances may also encompass diverse social factors, such as living arrangements with family or alone and workplace settings. These issues warrant attention when giving guidance on personalized radiation protection [34]. Patients should not be subjected to unreasonable restrictions either. However, they should be managed in a manner that complies with the ALARA (As Low As Reasonable Achievable) principle and ensures the protection of other individuals [4, 5, 34, 35]. Remote dose-rate monitoring can facilitate daily routines significantly when aiming at personalized radiation protection and optimizing the length of radiation isolation period. Ideally, patient’s comfort and staff efficiency are improved while generating economic savings for hospitals.

## Conclusion

The median whole-body T_eff_ of ^131^I for all patients was 12.60 h. Increased BMI, diabetes, and decreased GFR was found to prolong the whole-body T_eff_ of ^131^I in DTC patients. Ceiling-mounted remote DRMs provide reliable means to determine patient’s T_eff_. These findings could be utilized to optimize radiation protection and the length of radiation isolation during RAI therapies, paving the way for more personalized treatments.

## Data Availability

The data and materials are available on reasonable request to the corresponding author.
